# Serum OPN Expression for Identification of Gastric Cancer and Atrophic Gastritis and Its Influencing Factors

**DOI:** 10.1371/journal.pone.0114005

**Published:** 2014-12-05

**Authors:** Tiejun Chen, Liping Sun, Caiyun He, Yuehua Gong, Qian Xu, Yuan Yuan

**Affiliations:** 1 Tumor Etiology and Screening Department of Cancer Institute and General Surgery, the First Affiliated Hospital of China Medical University, and Key Laboratory of Cancer Etiology and Prevention (China Medical University), Liaoning Provincial Education Department, Shenyang 110001, China; 2 Medical Oncology Department of Benxi Central Hospital, Benxi, China; Second University of Naples, Italy

## Abstract

**Background:**

Most studies have found that osteopontin (OPN) expression level is related to the poor prognosis of gastric cancer. However, few studies have examined the relationship between OPN expression and gastric precancerous diseases, and the potential role of OPN in the formation and development of GC. We investigated the relationships between serum OPN levels and the risks of gastric cancer (GC) and its precancerous disease, to explore the diagnostic efficacy of serum OPN level for GC and atrophic gastritis and its influencing factors.

**Methods:**

A total of 1,452 patients were enrolled, including 609 with mild superficial gastritis (SG), 594 with atrophic gastritis (AG) and 249 with GC. The levels of serum OPN and serum *Helicobacter pylori* IgG antibody were detected by enzyme-linked immunosorbent assay.

**Results:**

Serum OPN levels increased from mild SG (1.99±1.91 ng/ml) to AG (2.37±2.27 ng/ml) to GC (5.94±4.52 ng/ml) (P≤0.002), along with increasing severity of gastric disease. OPN levels were significantly higher in patients with GC compared with the non-cancer population (2.17±2.10, P<0.0001). Serum OPN level was positively correlated with age and was higher in men than women, but was not correlated with *H. pylori* infection status. The area under the receiver operating characteristic curve was 0.805, the optimal cutoff was 2.56 ng/ml and the sensitivity and specificity were 74.3% and 71.8%, respectively, for the ability of serum OPN to discriminate GC.

**Conclusions:**

Serum OPN expression was closely related to the risks of GC and AG, and it might be a useful marker for the discrimination of GC. OPN level was positively correlated with age and male sex, but was not affected by *H. pylori* infection, and it was promoted by smoking and drinking, in patients with mild SG.

## Introduction

Gastric cancer (GC) is thought to occur via a stepwise progression from normal mucosa, through superficial gastritis (SG) and atrophic gastritis (AG) to carcinoma. During this process, AG and its possible accompanying changes, such as intestinal metaplasia and dysplasia, have been identified as gastric precancerous disease or precancerous lesions [1]. In some cases, the transition from precancerous disease to cancer is relatively reversible [2], [3], and early detection and treatment might therefore prevent the development of precancerous lesions into cancer, or allow the eradication of early cancers. However, the transition from precancerous disease to cancer is often hard to detect because of the inconspicuous symptoms, and most cancers are diagnosed at an advanced stage, making them hard to control or eradicate.

Valid screening and monitoring techniques for GC and precancerous diseases are thus urgently needed to decrease mortality, increase survival rates, and improve the prognosis of GC. Cancer screening and early diagnosis currently depend largely on barium X-ray examination, gastroscopic biopsies, and serological detection of tumor markers [4]. Detection of serum tumor markers has the advantages of being convenient, fast, and non-invasive, and has the potential to detect small changes before they become evident by imaging, or through the appearance of clinical symptoms. Serum tumor marker levels may also change according to the progression of the disease, and are thus able to played important roles in tumor screening, early diagnosis, surveillance and prognosis evaluation. However, the diagnostic performances of most serological tumor markers in terms of sensitivity and specificity are not ideal, which limits their clinical application and generalization. The identification of more effective serological tumor markers has thus become a major goal for GC screening and early diagnosis.

Osteopontin (OPN) is a secreted phosphorylated glycoprotein encoded by the SPP1 gene located on human chromosome 4q22.1. OPN is widely expressed in a variety of tissues, including bone, cartilage, kidney, blood vessels, skin and other tissues, and is also synthesized by various cell types, including macrophages, lymphocytes, and mammary epithelial cells, and can be detected in serum, urine, bile and milk [5]–[8]. OPN is a multifunctional protein, the major biological activities of which include regulating cell adhesion and chemotaxis, controlling cytokine expression, participating in cellular signal transduction, stimulating the cellular immunologic response and accelerating tissue remodeling [9]–[11]. OPN has recently been shown to be a component of the extracellular matrix, which plays a crucial role in tumor invasion, metastasis, apoptosis and angiogenesis [12]–[14], and high levels of OPN have been shown to correlate with higher clinical stage in many tumors [15], [16].

The relationship between OPN and GC has recently become a focus of interest. Most studies have found that OPN expression in GC tissues was significantly higher than in normal gastric tissues [14], [16]–[20], while serum OPN levels were also elevated in patients with GC [16]. There is strong evidence to indicate that higher levels of OPN expression in GC are associated with aggressive invasion, extensive metastasis and poorer prognosis [19], [21], [22]. However, few studies have examined the relationship between OPN expression and gastric precancerous diseases, and the potential role of OPN in the formation and development of GC. In addition, the factors influencing OPN expression remain unclear. It is therefore difficult to draw any firm conclusions about the relationship between serum OPN and the risk of GC and precancerous diseases. Furthermore, the efficiencies of serum OPN in discriminating gastric precancerous diseases and GC is uncertain.

In the present study, we measured serum OPN levels in a large population from northern China in order to explore the relationships between serum OPN and the risks of GC and precancerous diseases, and to clarify the factors affecting serum OPN levels. We also analyzed the values of serum OPN in discriminating GC and its precancerous diseases. These results will provide a valuable reference for the application of serum OPN in the discrimination of GC.

## Materials and Methods

### Subjects

This was a case-control study based on a population from northern China. A total of 1,452 patients with various gastric diseases was enrolled in the study. All subjects were recruited from GC screening of the general population in the Zhuanghe area of Liaoning Province, and from patients who were histologically certificated at the First Affiliated Hospital of China Medical University from 2002 to 2010. The diagnosis of GC was established by upper gastrointestinal endoscopic examination and confirmed by histopathology. Histopathologic findings were assessed according to the consensus on chronic gastritis formulated at the national symposium, or in combination with the visual analog scale of the updated Sydney System [23]. GC was diagnosed according to the WHO GC diagnosis criteria and the Lauren classification. Information on sex, age and other factors was obtained by means of a questionnaire administered to each patient. This study was approved by the Human Ethics Review Committee of the First Affiliated Hospital of China Medical University (Shenyang, China), and written informed consent was obtained from all patients, in accordance with the Declaration of Helsinki and its later revision.

Serum samples were collected from patients diagnosed with complete clinical pathology who agreed to provide blood samples, and for whom information on gender, age and relevant clinical data were available for analysis. Fasting blood serum was separated and stored at −20°C.

Information on smoking and alcohol consumption was collected by means of the questionnaire. A history of smoking was defined as daily smoking ≧1 cigarette for ≧1 year. A history of drinking was defined as an average daily alcohol intake ≧50 g and continued for ≧1 year.

### Detection of serum OPN

Approximately 5 ml fasting blood was collected from each participant and kept at 4°C for 24 h. The blood was centrifuged at 3500×*g* for 10 min and serum aliquots were stored immediately at −20°C, and then moved to −70°C prior to determination of various parameters. Serum OPN concentrations were measured by enzyme-linked immunosorbent assay (ELISA) using OPN ELISA kits (Wuhan Boster Biotechnology Company, Wuhan, Hubei, China), according to the manufacturer's protocol.

### Detection of serum *Helicobacter pylori* IgG antibody

Serum concentrations of *H. pylori* IgG were determined by ELISA (*H. pylori*-IgG ELISA kit, BIOHIT Plc, Helsinki, Finland), according to the manufacturer's protocol. Samples were considered positive when the serum *H. pylori* IgG antibody value was ≧34 units; other samples were considered negative.

### Statistical analysis

All statistical analyses were performed using SPSS 13.0 software (SPSS Inc., Chicago, IL, USA). Serum OPN expression was represented as mean ± standard deviation, and differences in serum OPN levels among groups, as well as in the stratified analysis, were compared by independent-sample *t*-tests and analysis of variance. Pearson's correlation coefficient was used to estimate the correlations between age, and *H. pylori* infection, and serum OPN levels. Receiver operating characteristic (ROC) curves and the area under the ROC curve (AUC) were used to evaluate the diagnostic effects of serum OPN and to determine appropriate cut-off points. A two-sided P value<0.05 was considered statistically significant.

## Results

### Characteristics of the study subjects

This study enrolled a total of 1,452 patients with various gastric diseases, including GC (n = 249), AG (n = 594) and mild SG, as a control (SG, n = 609). AG vs. mild SG: mean age 54 years; range 17–85 years; male/female 633/570; GC vs. mild SG: mean age 56 years; range 17–85 years; male/female 462/396. The rate of *H. pylori* infection was higher in AG (61.3%) and GC (54.6%) than SG (21.2%). The detailed baseline characteristics of the cases and controls including age,gender,smoking,drinking,*H. pylori* infection in our study are shown in Table 1.

**Table 1:** Characteristics of the study subjects.

Variables	Mild superficial gastritis	Atrophic gastritis	P*	Gastric cancer	P**
Total	n = 609	n = 594		n = 249	
Age(years): Mean±SD	53.10±9.86	55.03±8.94	0.000	58.40±11.42	0.000
Range	17–85	28–82		26–85	
Gender			0.006		0.000
Female	313(51.4%)	257(43.3%)		83(33.3%)	
Male	296(48.6%)	337(56.7%)		166(66.7%)	
Smoking			0.719		0.001
Smoker	131(28.9%)	143(30.0%)		56(44.4%)	
Non-smoker	323(71.1%)	333(70.0%)		70(55.6%)	
Drink			0.336		0.000
Drinker	90(19.8%)	107(22.5%)		48(38.4%)	
Nondrinker	364(80.2%)	369(77.5%)		77(61.6%)	
H. pylori infection status			0.000		0.000
HP(+)	129(21.2%)	364(61.3%)		136(54.6%)	
HP(−)	480(78.8%)	230(38.7%)		113(45.4%)	

P*: atrophic gastritis vs. mild superficial gastritis, P**: Gastric cancer vs. mild superficial gastritis.

### Correlations between serum OPN and gastric diseases

Serum OPN levels tended to increase from mild SG (1.99±1.91 ng/ml) to AG (2.37±2.27 ng/ml) to GC (5.94±4.52 ng/ml) (P≤0.002), in line with increasing severity of the gastric diseases. Serum OPN levels in AG were significantly higher than in mild SG (P = 0.002), were significantly higher in patients with GC compared with non-cancer subjects (2.17±2.10 ng/ml, P<0.0001). There were no significant differences in serum OPN levels between intestinal and diffuse-type GCs (P = 0.802) (Table 2).

**Table 2:** Levels of serum OPN (mean±SD) between different gastric disease groups.

	amount	OPN(ng/ml)	P-value
non-cancer	1203	2.17±2.10	
mild superficial gastritis	609	1.99±1.91	
atrophic gastritis	594	2.37±2.27	0.002*
gastric cancer	249	5.94±4.52	0.000^$#¥^
intestinal gastric cancer	85	5.58±3.80	
diffuse-type gastric cancer	110	5.74±5.02	0.802^&^

P *: atrophic gastritis vs. mild superficial gastritis, P^ $ # ¥^: Gastric cancer vs. mild superficial gastritis, atrophic gastritis and non-cancer respectively P ^&^: diffuse -type gastric cancer vs. intestinal cancer.

### Influence of age, sex, *H. pylori* infection, smoking and drinking on serum OPN

**Figure 1:**
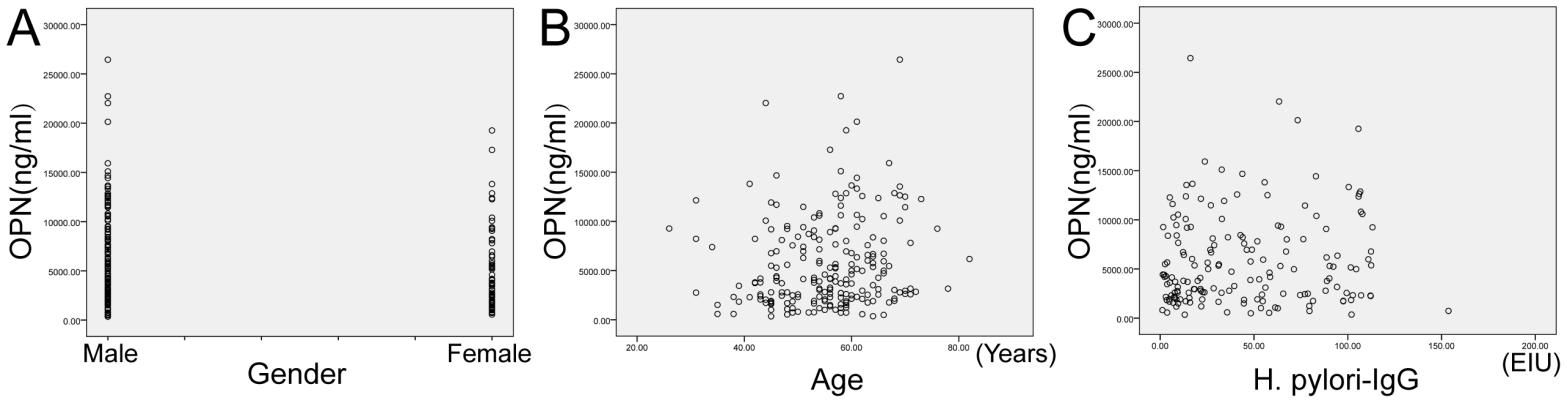
Scatter plots for the correlation between serum OPN and gender, age, H. pylori infection, in gastric cancer group. A, Gender; B, Age; C, H.pylori infection.

Patients were stratified by age, sex or *H. pylori* infection status (Table 3, Figure 1). In any group, the correlation coefficient for OPN and age (R1) was significantly above the 0 level (P<0.016), and the correlation coefficient for OPN and gender (R2) was significantly lower than the 0 level (P<0.049), Serum OPN level was increased with the age of the patients, and was higher in male sex than female sex. While there was no definite correlation between *H. pylori* infection and serum OPN, for the correlation coefficients for OPN and *H. pylori* (R3) were discrepant (P>0.108). Comparisons of serum OPN levels between men and women and between patients aged ≤50 years and >50 years in each disease group (Table 4) showed much higher serum OPN levels in men than women (P<0.013), and in the >50 year-old group compared with the ≤50 year-old group (P<0.024). However, there were no significant differences between the *H. pylori* IgG (+) group and the *H. pylori* IgG (−) group (P>0.113). Furthermore, serum OPN levels were elevated in smokers and drinkers (P<0.001, and P = 0.002, respectively) in the mild SG group compared with non-smokers and non-drinkers, but this discrepancy was not found in either the AG or GC group.

**Table 3:** Correlation between serum OPN levels and age, gender as well as H.pylori infection.

correlation coefficient	mild superficial gastritis (609)	atrophic gastritis (594)	gastric cancer (249)
R1 for OPN and age	0.115	0.135	0.154
p	0.004	0.001	0.016
R2 for OPN and gender	−0.09	−0.081	−0.171
P	0.027	0.049	0.007
R3 for OPN and H.pylori	0.023	−0.066	0.086
p	0.567	0.108	0.238

**Table 4:** Serum OPN expression in different gender, age, H. Pylori status, smoking and alcohol drinking.

	mild superficial gastritis (609)			atrophic gastritis (594)			gastric cancer (249)		
		OPN(ng/ml)	p		OPN(ng/ml)	p		OPN(ng/ml)	p
gender	male(296)	2.19±2.06	0.011	male (337)	2.57±2.26	0.013	male (166)	6.49±4.69	0.006
	female(313)	1.79±1.74		female(257)	2.10±2.27		female(83)	4.83±3.98	
age	≤50y(238)	1.60±1.47	0	≤50y(195)	2.01±1.59	0.002	≤50y(63)	4.83±4.26	0.024
	>50y(371)	2.24±2.11		>50y(399)	2.54±2.53		>50y(186)	6.31±4.56	
HP	HP+ (129)	2.13±1.93	0.352	HP+ (364)	2.33±2.36	0.639	HP+ (136)	6.35±4.77	0.113
	HP- (480)	1.95±1.90		HP- (230)	2.42±2.13		HP- (113)	5.44±4.17	
smoking	Smoker(131)	2.33±2.14	0	Smoker(143)	2.44±2.01	0.185	Smoker(56)	7.65±4.43	0.393
	Non (323)	1.51±1.36		Non (333)	2.16±2.19		Non (70)	6.98±4.36	
drinking	drinker(90)	2.35±2.17	0.002	drinker(107)	2.31±1.92	0.706	drinker(48)	7.98±4.66	0.164
	Non (364)	1.60±1.48		Non (369)	2.22±2.20		Non (77)	6.85±4.20	

### Serum OPN in gastric diseases, adjusted for age and sex

Stratification analysis showed that, even after adjusting for age (50 years) and sex, serum OPN levels still tended to increase along with the seriousness of the gastric diseases from mild SG to AG to GC. However, the difference in serum OPN levels between the mild SG and AG groups were less evident in most subgroups, except for the ≤50-year-old men subgroup (P = 0.009). Meanwhile, serum OPN levels in the GC group were obviously higher than in the mild SG and AG groups (P≤0.002 and ≤0.004, respectively) (Table 5).

**Table 5:** Serum OPN expression in gastric diseases, adjusted for age and gender.

gender	age	Gastric disease	amount	OPN(ng/ml)	p
		mild superficial gastritis	94	1.79±1.39	
	≤50y	atrophic gastritis	89	2.42±1.84	0.009*
male		gastric cancer	39	5.39±4.69	0.000^$#^
		mild superficial gastritis	202	2.38±2.28	
	>50y	atrophic gastritis	248	2.62±2.40	0.272*
		gastric cancer	127	6.83±4.65	0.000^$#^
		mild superficial gastritis	144	1.47±1.51	
	≤50y	atrophic gastritis	106	1.67±1.26	0.275*
female		gastric cancer	24	3.91±3.34	0.002^$^, 0.004^#^
		mild superficial gastritis	169	2.07±1.87	
	>50y	atrophic gastritis	151	2.40±2.73	0.196*
		gastric cancer	59	5.20±4.18	0.000^$#^

P*: atrophic gastritis vs. mild superficial gastritis, P^$ #^: gastric cancer vs. mild superficial gastritis, atrophic gastritis respectively.

### Efficiency of serum OPN for discriminating AG

ROC curves were plotted for serum OPN to discriminate gastric diseases from normal stomach. Overall, the efficiency of serum OPN for discriminating AG was weak; although the value was significant (P = 0.002), the AUC was 0.555 and the best cut-off point for serum OPN was 1.33 ng/ml, with corresponding validity parameters of 61.6% sensitivity and 49.4% specificity. Stratification analysis showed that the efficiency of serum OPN for discriminating AG was only significant in patients 50 years or under (≤50-year-old men, P = 0.001; ≤50-year-old women, P = 0.024) (Table 6).

**Table 6:** Efficiency of serum OPN in discriminating atrophic gastritis.

	gender	age	best cut-off	sensitivity	specificity	YD	area under curve	P
			(ng/ml)					
total			1.33	0.616	0.494	0.11	0.555	0.002
	male	≤50y	1.33	0.694	0.606	0.3	0.648	0.001
		>50y	1.906	0.516	0.576	0.092	0.533	0.24
	female	≤50y	0.521	0.934	0.283	0.217	0.591	0.024
		>50y	1.41	0.608	0.479	0.087	0.52	0.548

### Efficiency of serum OPN for discriminating GC

**Figure 2:**
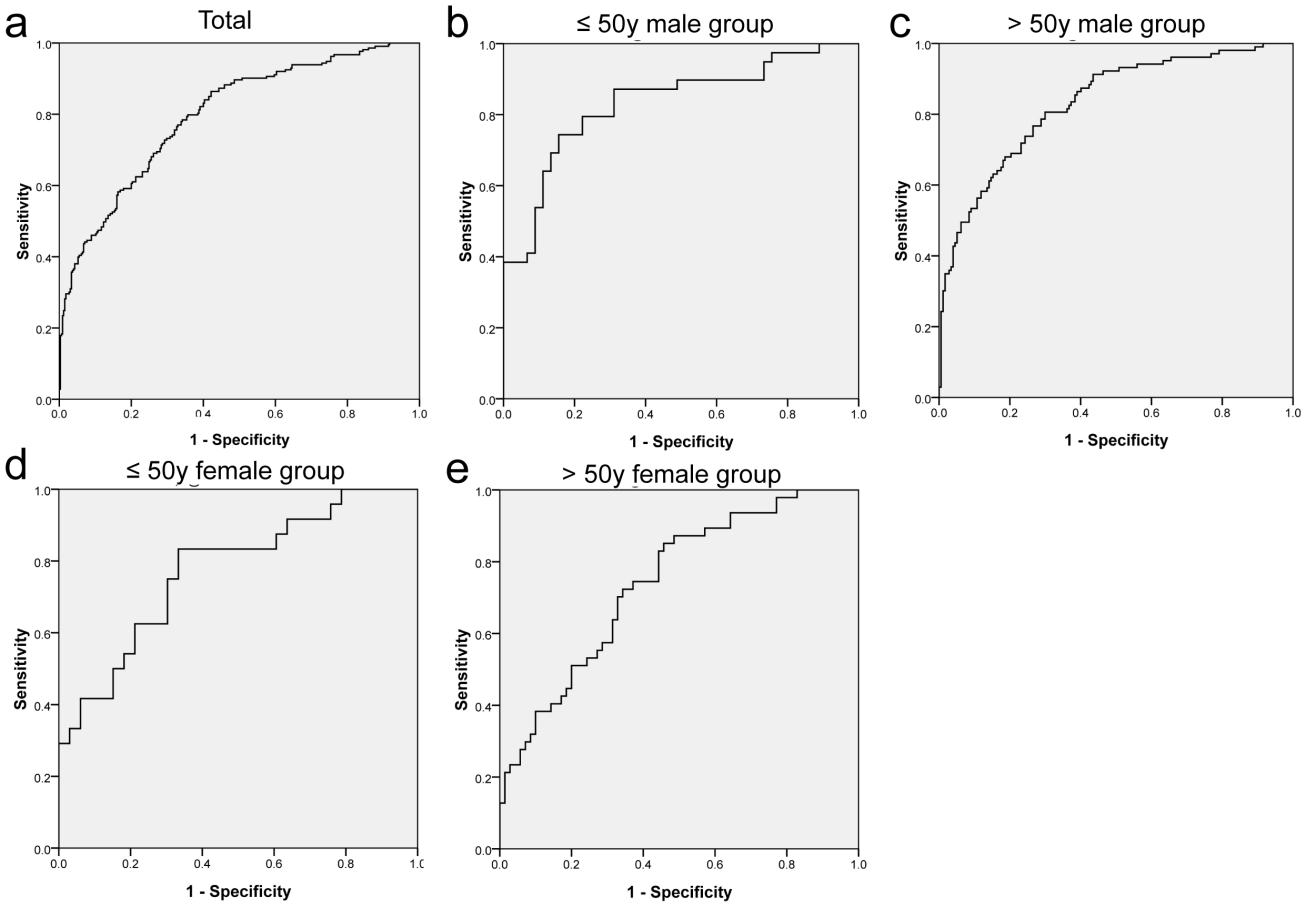
ROC curve for serum OPN levels to discriminate gastric cancer. a, total; b, ≤50y male group; c, >50y male group; d, ≤50y female group; e, >50y female group.

ROC curves indicated that serum OPN showed significant efficiency for discriminating GC (P<0.001) (Table 7). In total, the AUC was 0.805 and the best cut-off point for serum OPN was 2.56 ng/ml, with corresponding validity parameters of 74.3% sensitivity and 71.8% specificity. In stratification analysis, serum OPN also showed significant ability to recognize GC in all subgroups (P<0.001). The AUC in the ≤50-year-old men group was 0.746, and the best cut-off point for serum OPN was 2.43 ng/ml, with corresponding validity parameters of 71.8% sensitivity and 66.7% specificity. The equivalent AUC in the >50-year-old men group was 0.833, and the best cut-off point for serum OPN was 2.68 ng/ml, with corresponding validity parameters of 84.3% sensitivity and 68.4% specificity. The AUC in the ≤50-year-old women group was 0.786, and the best cut-off point for serum OPN was 1.71 ng/ml, with corresponding validity parameters of 83.3% sensitivity and 68.8% specificity, and the equivalent AUC in the >50-year-old women group was 0.762, the best cut-off point for serum OPN was 2.48 ng/ml, and the corresponding validity parameters were 72.9% sensitivity and 70.0% specificity (Table 7, Figure 2).

**Table 7:** Efficiency of serum OPN in discriminating gastric cancer.

	gender	age	best cut-off	sensitivity	specificity	YD	area under curve	P
			(ng/ml)					
total			2.56	0.743	0.718	0.461	0.805	0.000
	male	≤50y	2.427	0.718	0.667	0.385	0.746	0.000
		>50y	2.68	0.843	0.684	0.527	0.833	0.000
	female	≤50y	1.71	0.833	0.688	0.521	0.786	0.000
		>50y	2.48	0.729	0.7	0.429	0.762	0.000

## Discussion

Most studies have found that OPN expression level is related to the poor prognosis of gastric cancer. However, few studies focused on the potential role of OPN in the formation of GC. In the present study, we investigated the relationship between serum OPN level and the risks of gastric cancer (GC) and its precancerous disease to further explore the diagnostic efficacy of serum OPN level for GC and atrophic gastritis and its influencing factors. Our results revealed that serum OPN expression was closely related to the risks of GC and AG, and was positively correlated with age and male, and was promoted by smoking and drinking in patients with mild SG. To the best of our knowledge, the current study provides the most explicit report indicating that serum OPN level may play an important role not only in the gastric cancer progression, but also in its formation, and serum OPN level may have potential usefulness as a screening or diagnostic factor for early gastric cancer.

As many studies have revealed, OPN status was significantly associated with gastric cancer development, invasive phenotypes, shorter survival time and poor prognosis [14], [16], [24]. Nevertheless, the clinical significance of serum OPN expression in estimating gastric cancer risk remains unclear. Atrophy gastritis has been identified as a major gastric precancerous lesion [25]. Early detection of this precancerous lesion may therefore help to prevent gastric cancer. Up to now, few studies have concerned on the usefulness of serum OPN expression in atrophy gastritis [26]. We measured the serum OPN expression by ELISA in a large population from northern China, and found that serum OPN level increased with worsening of gastric diseases, from mild SG to AG to GC. Serum OPN level in the GC group remained obviously higher than in the mild SG and AG groups. To clarify the clinical application value of serum OPN levels in gastric diseases, we explored its efficacies for discriminating GC and AG. Based on ROC curves, serum OPN demonstrated significant efficacy for recognizing GC. The AUCs, cut-off points for serum OPN, and the sensitivity and specificity were compatible with its use for discriminating GC, both in the overall population and in patients stratified according to age (50 years) or sex. Our results thus suggested that serum OPN level was an indicator of severity in gastric diseases, and serum OPN may be a sensitive biomarker for monitoring and predicting GC risk.

Previous studies have shown that the serum OPN levels can be affected by various factors, including age, sex, infections, smoking and alcohol. However, the exact natures of these influences remain elusive. In this study, serum OPN levels were positively correlated with patient age and were higher in patients >50 years, at all levels of diseases. These results were consistent with those of other studies [14], [27]. It is likely that the higher OPN levels in elderly patients were mostly caused by higher expression levels in macrophages, rather than by an increasing number of infiltrated macrophages. However, in contrast with these results, some studies failed to find any relationship between OPN levels and age [20], [28], and more studies are therefore needed to clarify this issue.

We also found a significant difference in serum OPN levels between men and women; OPN levels were significantly higher in men in the mild SG, AG and GC groups. Thus, we suspect female hormones may have some effect on OPN expression. But previous studies indicated that this effect is complex. Estrogen may play different roles in diverse types of cells [29]. For example, in invasive breast cancer tissues, estrogen was negatively correlated with OPN expression [30], but other study suggested estrogen increased OPN level [31]. Furthermore, different doses of estradiol also exert different effects on OPN levels [32]: low-dose estradiol may down-regulate the expression of OPN mRNA, while high-dose estradiol can up-regulate it. Some recent studies [33]–[35] found that serum OPN levels had a significant negative correlation with bone mineral density in menopausal women, and it was considered a negative feedback effect on bone mediated by OPN through beta-adrenergic signal pathway [36]. These findings may help us better understand the relationship between serum OPN and age and sex, though the precise mechanism remains unclear.

The influence of *H. pylori* infection on serum OPN levels has rarely been reported. In this study, we found no significant differences in OPN levels between *H. pylori* IgG (+) and *H. pylori* IgG (−) patients with different gastric diseases. Researchers in Taiwan [37] found that OPN expression levels were elevated in *H. pylori*-infected gastric mucosa compared with non-infected mucosa, and the heavier the inflammation of the stomach, the higher level of OPN expressed in gastric mucosa. They further confirmed that the OPN expressed in the gastric mucosa was produced primarily by monocytes, rather than by gastric epithelial cells. It has been reported [38] that increased OPN expression was closely related to infiltration of monocytes macrophages and increased cellular proliferation, in renal tubules. But it remains unclear whether the relationship between OPN levels and monocyte infiltration also exists in gastric mucosa. Further studies are therefore needed to elucidate this relationship.

The results of the current study showed that both smoking and drinking significantly promoted serum OPN expression, at least in patients with mild SG. This finding has been supported by many studies [39]–[42]. It has been proven that both nicotine and alcohol give rise to elevated OPN level. Several early studies [43]–[45] had revealed that smoking and alcohol have some effect on chemokine mRNA expression, in the progression of chronic gastritis to atrophy. In this study, we found that the effects of smoking and drinking on OPN expression in patients with mild SG were not replicated in patients with AG or GC, so we speculate that the influences of smoking and drinking on GC risk may lie principally in the process of transition from SG to AG, rather than the subsequent transition from AG to GC, and OPN may play an important role in the process of chronic gastritis to atrophy, despite the lack of published evidence. If our hypothesis is confirmed, it suggests that more emphasis should be placed on smoking and drinking cessation in patients with gastric diseases prior to the development of AG.

This study had several limitations. First, *H. pylori* status was only judged by serum IgG anti-*H. pylori* rather than using more effective test such as 13C Urea Breath Test or HpSA in stool. Second,there is no OLGA score available to determine the risk of development of gastric cancer in the patients with atrophic gastritis. Third, the relationships between OPN expression and smoking and drinking were not examined in depth because of inadequate data on cigarette use and alcohol consumption. We were unable to elaborate on the correlation between serum OPN levels and different types of GCs, because of limited information on GCs. In addition, histological samples and OPN tissue expression were not investigated in this study. Further studies based on more complete data are therefore warranted to verify and supplement the results of the current study.

In conclusion, the results of this study suggest that serum OPN expression was closely related to the risks of GC and AG, and it might be a useful marker for the discrimination of GC. OPN level was positively correlated with age and male, but was not affected by *H. pylori* infection, and it was promoted by smoking and drinking in patients with mild SG. So, close attention should be paid to changes in serum OPN levels in patients already diagnosed with AG. After adjusting for age and sex, serum OPN may be an effective reference for the early recognition of GC, and may provide a useful guidance for choosing appropriate treatment measures for patients with gastric diseases.

## References

[1] Dinis-Ribeiro M, Areia M, de Vries AC, Marcos-Pinto R, Monteiro-Soares M, et al (2012) Management of precancerous conditions and lesions in the stomach (MAPS): guideline from the European Society of Gastrointestinal Endoscopy (ESGE), European Helicobacter Study Group (EHSG), European Society of Pathology (ESP), and the Sociedade Portuguesa de Endoscopia Digestiva (SPED). Endoscopy 44:74–94. 22198778 10.1055/s-0031-1291491 PMC3367502

[2] Wijkstrom H, Cohen SM, Gardiner RA, Kakizoe T, Schoenberg M, et al. (2000) Prevention and treatment of urothelial premalignant and malignant lesions. Scand J Urol Nephrol Suppl: 116–135. 10.1080/00365590050509878 11144892

[3] Gustafson AM, Soldi R, Anderlind C, Scholand MB, Qian J, et al (2010) Airway PI3K pathway activation is an early and reversible event in lung cancer development. Sci Transl Med 2:26ra25. 10.1126/scitranslmed.3000251 PMC3694402 20375364

[4] Yuan Y (2013) A survey and evaluation of population-based screening for gastric cancer. Cancer Biol Med 10:72–80. 23882421 10.7497/j.issn.2095-3941.2013.02.002 PMC3719193

[5] Chen YJ, Shen JL, Wu CY, Chang YT, Chen CM, et al (2009) Elevated plasma osteopontin level is associated with occurrence of psoriasis and is an unfavorable cardiovascular risk factor in patients with psoriasis. J Am Acad Dermatol 60:225–230. 19028408 10.1016/j.jaad.2008.09.046

[6] Wasilewska A, Taranta-Janusz K, Kuroczycka-Saniutycz E, Zoch-Zwierz W (2011) Urinary OPN excretion in children with glomerular proteinuria. Adv Med Sci 56:193–199. 21983451 10.2478/v10039-011-0034-y

[7] Schack L, Lange A, Kelsen J, Agnholt J, Christensen B, et al (2009) Considerable variation in the concentration of osteopontin in human milk, bovine milk, and infant formulas. J Dairy Sci 92:5378–5385. 19841198 10.3168/jds.2009-2360

[8] Imano M, Satou T, Itoh T, Takeyama Y, Yasuda A, et al (2010) An immunohistochemical study of osteopontin in pigment gallstone formation. Am Surg 76:91–95. 20135947

[9] Rangaswami H, Bulbule A, Kundu GC (2006) Osteopontin: role in cell signaling and cancer progression. Trends Cell Biol 16:79–87. 16406521 10.1016/j.tcb.2005.12.005

[10] Chakraborty G, Jain S, Behera R, Ahmed M, Sharma P, et al (2006) The multifaceted roles of osteopontin in cell signaling, tumor progression and angiogenesis. Curr Mol Med 6:819–830. 17168734 10.2174/156652406779010803

[11] El-Tanani MK (2008) Role of osteopontin in cellular signaling and metastatic phenotype. Front Biosci 13:4276–4284. 18508510 10.2741/3004

[12] Anborgh PH, Mutrie JC, Tuck AB, Chambers AF (2010) Role of the metastasis-promoting protein osteopontin in the tumour microenvironment. J Cell Mol Med 14:2037–2044. 20597997 10.1111/j.1582-4934.2010.01115.x PMC3822994

[13] Takafuji V, Forgues M, Unsworth E, Goldsmith P, Wang XW (2007) An osteopontin fragment is essential for tumor cell invasion in hepatocellular carcinoma. Oncogene 26:6361–6371. 17452979 10.1038/sj.onc.1210463

[14] Higashiyama M, Ito T, Tanaka E, Shimada Y (2007) Prognostic significance of osteopontin expression in human gastric carcinoma. Ann Surg Oncol 14:3419–3427. 17896150 10.1245/s10434-007-9564-8

[15] Blasberg JD, Pass HI, Goparaju CM, Flores RM, Lee S, et al (2010) Reduction of elevated plasma osteopontin levels with resection of non-small-cell lung cancer. J Clin Oncol 28:936–941. 20085934 10.1200/JCO.2009.25.5711 PMC2834433

[16] Wu CY, Wu MS, Chiang EP, Wu CC, Chen YJ, et al (2007) Elevated plasma osteopontin associated with gastric cancer development, invasion and survival. Gut 56:782–789. 17148500 10.1136/gut.2006.109868 PMC1954839

[17] Junnila S, Kokkola A, Mizuguchi T, Hirata K, Karjalainen-Lindsberg ML, et al (2010) Gene expression analysis identifies over-expression of CXCL1, SPARC, SPP1, and SULF1 in gastric cancer. Genes Chromosomes Cancer 49:28–39. 19780053 10.1002/gcc.20715

[18] Dai N, Bao Q, Lu A, Li J (2007) Protein expression of osteopontin in tumor tissues is an independent prognostic indicator in gastric cancer. Oncology 72:89–96. 18004082 10.1159/000111108

[19] Imano M, Satou T, Itoh T, Sakai K, Ishimaru E, et al (2009) Immunohistochemical expression of osteopontin in gastric cancer. J Gastrointest Surg 13:1577–1582. 19582521 10.1007/s11605-009-0955-y

[20] Zhang X, Tsukamoto T, Mizoshita T, Ban H, Suzuki H, et al (2009) Expression of osteopontin and CDX2: indications of phenotypes and prognosis in advanced gastric cancer. Oncol Rep 21:609–613. 19212618

[21] Song G, Ouyang G, Mao Y, Ming Y, Bao S, et al (2009) Osteopontin promotes gastric cancer metastasis by augmenting cell survival and invasion through Akt-mediated HIF-1alpha up-regulation and MMP9 activation. J Cell Mol Med 13:1706–1718. 19602039 10.1111/j.1582-4934.2008.00540.x PMC6512381

[22] Tang H, Wang J, Bai F, Zhai H, Gao J, et al (2008) Positive correlation of osteopontin, cyclooxygenase-2 and vascular endothelial growth factor in gastric cancer. Cancer Invest 26:60–67. 18181047 10.1080/07357900701519279

[23] Dixon MF, Genta RM, Yardley JH, Correa P (1996) Classification and grading of gastritis. The updated Sydney System. International Workshop on the Histopathology of Gastritis, Houston 1994. Am J Surg Pathol 20:1161–1181. 8827022 10.1097/00000478-199610000-00001

[24] Sun XJ, Zuo WS, Ma H, Hou WH, Cai SP, et al (2005) [Expression of osteopontin mRNA and its clinical significance in gastric cancer]. Zhonghua Zhong Liu Za Zhi 27:292–295. 15996323

[25] Dirnu R, Secureanu FA, Neamtu C, Totolici BD, Pop OT, et al (2012) Chronic gastritis with intestinal metaplasia: clinico-statistical, histological and immunohistochemical study. Rom J Morphol Embryol 53:293–297. 22732798

[26] Gong YH, Sun LP, Yuan Y (2006) [Evaluation of serum pepsinogen I, II and osteopontin co-detection in gastric cancer screening]. Zhonghua Zhong Liu Za Zhi 28:691–693. 17274377

[27] Paliwal P, Pishesha N, Wijaya D, Conboy IM (2012) Age dependent increase in the levels of osteopontin inhibits skeletal muscle regeneration. Aging (Albany NY) 4:553–566. 22915705 10.18632/aging.100477 PMC3461343

[28] Cristaudo A, Foddis R, Bonotti A, Simonini S, Vivaldi A, et al (2010) Comparison between plasma and serum osteopontin levels: usefulness in diagnosis of epithelial malignant pleural mesothelioma. Int J Biol Markers 25:164–170. 20878622 10.1177/172460081002500307

[29] Zirngibl RA, Chan JS, Aubin JE (2008) Estrogen receptor-related receptor alpha (ERRalpha) regulates osteopontin expression through a non-canonical ERRalpha response element in a cell context-dependent manner. J Mol Endocrinol 40:61–73. 18234909 10.1677/JME-07-0114

[30] Ribeiro-Silva A, Oliveira da Costa JP (2008) Osteopontin expression according to molecular profile of invasive breast cancer: a clinicopathological and immunohistochemical study. Int J Biol Markers 23:154–160. 18949741 10.1177/172460080802300304

[31] Miyajima J, Hayashi T, Saito K, Iida S, Matsuoka K (2010) The Interaction between female sex hormone receptors and osteopontin in a rat hyperoxaluric model. Kurume Med J 57:73–80. 21186342 10.2739/kurumemedj.57.73

[32] Banerjee A, Rose R, Johnson GA, Burghardt RC, Ramaiah SK (2009) The influence of estrogen on hepatobiliary osteopontin (SPP1) expression in a female rodent model of alcoholic steatohepatitis. Toxicol Pathol 37:492–501. 19387089 10.1177/0192623309335633

[33] Chang IC, Chiang TI, Yeh KT, Lee H, Cheng YW (2010) Increased serum osteopontin is a risk factor for osteoporosis in menopausal women. Osteoporos Int 21:1401–1409. 20238102 10.1007/s00198-009-1107-7

[34] Cho EH, Cho KH, Lee HA, Kim SW (2013) High serum osteopontin levels are associated with low bone mineral density in postmenopausal women. J Korean Med Sci 28:1496–1499. 24133355 10.3346/jkms.2013.28.10.1496 PMC3792605

[35] Fodor D, Bondor C, Albu A, Simon SP, Craciun A, et al (2013) The value of osteopontin in the assessment of bone mineral density status in postmenopausal women. J Investig Med 61:15–21. 10.2310/JIM.0b013e3182761264 23117696

[36] Farr JN, Charkoudian N, Barnes JN, Monroe DG, McCready LK, et al (2012) Relationship of sympathetic activity to bone microstructure, turnover, and plasma osteopontin levels in women. J Clin Endocrinol Metab 97:4219–4227. 22948767 10.1210/jc.2012-2381 PMC3485606

[37] Chang WL, Yang HB, Cheng HC, Chuang CH, Lu PJ, et al (2011) Increased gastric osteopontin expression by Helicobacter pylori Infection can correlate with more severe gastric inflammation and intestinal metaplasia. Helicobacter 16:217–224. 21585607 10.1111/j.1523-5378.2011.00832.x

[38] Cao Z, Cox A, Bonnet F (2002) Increased osteopontin expression following renal ablation is attenuated by angiotensin type 1 receptor antagonism. Exp Nephrol 10:19–25. 11803201 10.1159/000049894

[39] Hillas G, Loukides S, Kostikas K, Simoes D, Petta V, et al (2013) Increased levels of osteopontin in sputum supernatant of smoking asthmatics. Cytokine 61:251–255. 23098767 10.1016/j.cyto.2012.10.002

[40] Sullivan J, Blair L, Alnajar A, Aziz T, Chipitsyna G, et al (2011) Expression and regulation of nicotine receptor and osteopontin isoforms in human pancreatic ductal adenocarcinoma. Histol Histopathol 26:893–904. 21630219 10.14670/HH-26.893

[41] Patouraux S, Bonnafous S, Voican CS, Anty R, Saint-Paul MC, et al (2012) The osteopontin level in liver, adipose tissue and serum is correlated with fibrosis in patients with alcoholic liver disease. PLoS One 7:e35612. 22530059 10.1371/journal.pone.0035612 PMC3329460

[42] Kong LB, Ren WG, Mi HM, Zhao SX, Zhang YG, et al (2013) [Establishment of a complex alcoholic liver fibrosis mouse model and investigation of OPN and TGF-beta1 hepatic expression]. Zhonghua Gan Zang Bing Za Zhi 21:207–212. 23967743

[43] Shimoyama T, Everett SM, Fukuda S, Axon AT, Dixon MF, et al (2001) Influence of smoking and alcohol on gastric chemokine mRNA expression in patients with Helicobacter pylori infection. J Clin Pathol 54:332–334. 11304855 10.1136/jcp.54.4.332 PMC1731392

[44] Negrini R, Savio A, Poiesi C, Appelmelk BJ, Buffoli F, et al (1996) Antigenic mimicry between Helicobacter pylori and gastric mucosa in the pathogenesis of body atrophic gastritis. Gastroenterology 111:655–665. 8780570 10.1053/gast.1996.v111.pm8780570

[45] Sakaki N, Kozawa H, Egawa N, Tu Y, Sanaka M (2002) Ten-year prospective follow-up study on the relationship between Helicobacter pylori infection and progression of atrophic gastritis, particularly assessed by endoscopic findings. Aliment Pharmacol Ther 16 Suppl 2: 198–203. 11966542 10.1046/j.1365-2036.16.s2.13.x

